# Influence of Stacking Sequence on Mechanical Properties of Basalt/Ramie Biodegradable Hybrid Polymer Composites

**DOI:** 10.3390/polym15040985

**Published:** 2023-02-16

**Authors:** Velumayil Ramesh, Krishnasamy Karthik, Robert Cep, Muniyandy Elangovan

**Affiliations:** 1 Department of Mechanical Engineering, Vel Tech Rangarajan Dr. Sagunthala R&D Institute of Science and Technology, Avadi 600 062, India; 2 Department of Machining, Assembly and Engineering Metrology, Faculty of Mechanical Engineering, VSB-Technical University of Ostrava, 17. Listopadu 2172/15, 708 00 Ostrava, Czech Republic; 3 Department of R&D, Bond Marine Consultancy, London EC1V 2NX, UK

**Keywords:** tensile, flexural, hybrid composite, impact and hardness, SEM, microstructure analysis

## Abstract

In this study, the mechanical properties of basalt/ramie/polyester hybrid composite laminates were investigated. A matrix of 45% polyester was used, as it has good bonding properties between fibers. The composite laminates were fabricated using a hand layup technique, with seven layers stacked in different sequences and impregnated in the polyester matrix to create a hybrid configuration. Tensile, flexural, impact, compression, and hardness tests were conducted according to ASTM standards for mechanical characterization. The results showed that the overall stacking sequence of sample number seven (BRBRBRB) had the highest tensile strength at 120 MPa, impact energy at 8 J, flexural strength at 115 MPa, compression strength at 70 MPa, and hardness of 77. Natural fiber-reinforced composites are being used in current automotive industry applications, such as in electric vehicles.

## 1. Introduction

Many researchers are developing natural fiber-reinforced composite (NFRC) materials by extracting them from plants. These materials have several advantages, including being readily available, reproducible, biodegradable, non-toxic, and environmentally friendly. They are also recyclable and sustainable, making them popular in the automotive industry for use in electric vehicles. Natural composite materials can be found in plants and animals. For example, wood is made up of long cellulose fibers, a type of polymer, held together by the weaker component lignin. Cotton also contains cellulose, but it is in a more fragile state because it lacks the lignin that holds wood fibers together. The combination of lignin and cellulose creates a more robust material. Additionally, bones in the human body are a composite material, consisting of hydroxyapatite, mainly composed of calcium phosphate, and collagen, a protein that is both soft and flexible. An early example of a natural composite material is a mud brick. By allowing mud to dry into the shape of a brick, a building material with good compressive strength can be created. However, it has poor tensile strength and will shatter easily if bent. By combining straw and mud to create a mixture, bricks with improved resistance to squeezing and tearing can be made. These bricks are suitable for use as building blocks [1,2,3].

In recent years, fiber-reinforced polymer composite materials made from natural plant fibers have gained popularity due to their superior properties compared to artificial fibers. These natural fibers, which are abundant in India’s forests and villages, are used in a variety of day-to-day applications including the production of plates, cups, coir threads, ladders, and other products made from coconut, banana, and bamboo trees [4]. According to Ganesan et al. [5], it is expected that the trend will move away from using plastic and towards using hybrid composite materials in the future. A literature review was conducted to identify the resources used and areas where there are gaps in current knowledge. Several mechanical characterization methods, including tensile testing, impact testing, flexural testing, and dynamic mechanical analysis, were used to examine the performance of hybrid polymer matrix composites (HPMCs). Using a projection approach, the relationship between fiber density and properties such as Young’s modulus and flexural strength was determined in hemp Crete, a combination of hemp and lime. It was found that increasing the composite’s density improved its mechanical and thermal properties [6].

Karthik et al. [7] researched the use of glass fiber-reinforced polymer matrix composites as a potential replacement material for use in automobiles and airplanes due to their low weight and high strength. The hand-laying method used to prepare the specimens is described in the article, and the tensile, hardness and impact energy characteristics were characterized according to the American Society for Testing and Materials (ASTM) standards. The study used glass woven fabric as the reinforcing medium and polyester resin as the matrix material, both of which are readily available. Mohanavel et al. [8] investigated the effect of fiber composition, layering arrangement, and sequencing on the mechanical characteristics of polymer composites. Four specific matrix composites with five laminae and varied layering patterns of jute/Madar/glass fibers were prepared using a manual method. Yoganandam et al. [9] studied the effect of ageing on saltwater and hand-laid-up composites kept at room temperature for 90 days. The results showed that the hybrid composite’s flexural strength was highest (490 MPa) when it was dry. In comparison to carbon composite reinforced with plain glass fiber, the hybrid composite exhibited 44% lower diffusivity in saltwater.

In recent years, global researchers have focused on enhancing the mechanical properties of natural fiber-reinforced composites using various techniques, such as pinning, stitching, and the addition of short and long fibers [10]. The stacking sequence of fibers and different weight percentages of reinforcement are varied to develop new composite laminates, as studied by Nagamadhu et al. [11]. Basalt fibers, derived from naturally occurring volcanic rocks with finely grained fibers with widths ranging from 10 to 20 μm, have been investigated by Abhilash et al. [12] for their favorable characteristics, including high heat conductivity, high elongation, and low density, although they also have relatively poor thermal conductivity and find various applications in textiles, insulation, and heat-resistant plates. Hybrid fiber-reinforced composites, which combine the benefits of multiple fibers, are a significant advancement over using individual fibers [13]. For example, pure ramie fibers have a tensile strength of 50–60 MPa but the tensile strength of basalt fibers is around 100 MPa. The mechanical characteristics of hybrid composite laminates can be improved by combining these two fibers and employing a different stacking sequence during fabrication, as reported by several studies [14,15,16]. In addition, the incorporation of Kevlar fibers into a composite made of natural fibers can improve its mechanical properties to a greater extent than using natural fibers alone. In this study, we use a saturated polyester matrix to construct four distinct laminates made of basalt and ramie mixed fibers stacked in an alternating six-layer sequence. These hybrid composite materials have potential applications in roofing and other structural uses [17,18].

There was a variation in the tensile mechanical characteristics of a polymer composite reinforced with basalt fiber with changing yield stress and temperature levels. The digital image correlation (DIC) approach generated whole field distortion distributions in basalt fiber reinforced polymer (BFRP) specimens, which accurately reflected the composite material’s characteristic non-uniform pattern. The DIC-determined strain responses were then averaged, and the experimental stressors were associated with them. The mechanical properties of natural fiber-reinforced polymer composites (NPCs) have been studied extensively with regard to different strain rates, as demonstrated by the extensive literature on the topic. This review summarizes what has been learned about the approaches to characterizing strain rates, but also reveals that there are still obstacles to overcome when describing mechanical properties over a broad range of strain velocities [19,20]. Quagliarini et al. [21] investigated the durability of basalt fiber ropes and rods for structural engineering, as these are some of the newest popular basalt products in civil engineering and construction. They subjected these products to durability tests and found that, while basalt is a sturdy material, the literature on the durability of basalt products is mixed at best.

Prasad and Talupula [22] reviewed research on the use of basalt and aramid (Kevlar 129) fibers for reinforcement. They found that using a hybrid composite of basalt fiber reinforced with Kevlar could potentially save costs and improve water resistance through the use of compatibility and bonding promoters. While Kevlar has high quality, it also has several drawbacks including low compressive strength and moisture absorption. To address these issues, a hybrid composite of basalt fiber and epoxy resin was suggested. Natural fibers such as basalt are being chosen to produce low-cost and lightweight composites due to the increasing demand for sustainable and environmentally friendly products. This paper provides an overview of the chemistry of basalt and Kevlar.

Das et al. [23] found that plain glass/polyester (S7) had the best mechanical properties of all the tested materials, with tensile, bending and flexural strength 146.3, 167.6, and 188.7% stronger than that of neat jute/polyester (S0). The material characteristics of composite samples are largely influenced by the stacking sequence and the quantity and location of glass fiber layers in the laminated composites. The mechanical properties were highest in S6, a hybrid composite, compared to the others. Khalid et al. [24] emphasized the importance of finding entirely combustible or biodegradable materials to reduce the environmental impact of high-tech materials, a vital and challenging task. They argued that natural fiber-reinforced polymer composites (NFRPCs) are a better choice than synthetic fiber-reinforced composites due to their abundance, cost-effectiveness, and potential to improve physical and mechanical properties through chemical treatments, coatings, and hybridization processes. This paper also discusses the synthesis, categorization, characterization, and applications of responsive composite microgels made with silver nanoparticles.

The mechanical properties of glass/Kevlar hybrid composite laminated sheets were studied with varying fiber in-plane alignments and stacking sequences. The mechanical characterization was performed using tension and three-point bend tests, along with numerical simulation, to investigate the mechanical characteristics and damage mechanism. Additionally, the flax-Kevlar hybrid epoxy composite and its mechanism of deterioration were analyzed.

This article summarizes the results obtained using fiber-reinforced composites produced by additive manufacturing (AM), specifically the fused deposition modeling (FDM) technique. The FDM method has recently made significant progress in producing fiber composites, and all types of fiber-reinforced composites, including those made with short and long fibers, were analyzed in this review. While the impact of carbon fiber reinforcement on FDM-fabricated composites has received the most attention, future studies should investigate the impact of other natural and synthetic fiber reinforcements. Few researchers have looked at the impact of natural fibers on FDM, which has led to the development of biocomposites using this technique. Some researchers focused on extracting fibers from plants using various techniques, and after extracting long and short fibers they used them in a variety of applications, including those in the automotive industry and the domestic/homemade product sector, highlighting the importance of the existing literature. The mechanics of basalt-reinforced polymer composites and the role of hybrid glass/Kevlar fibers have also been studied. Natural fibers are increasingly being considered as reinforcements for polymer composites due to their environmental and cost-effective advantages, but their full potential as reinforcements remains untapped until certain limitations, such as lower strength and greater water absorption capacity than inorganic fibers, are addressed. Combining two or more fibers, such as natural and synthetic fibers, can easily improve these characteristics.

## 2. Materials and Methods

### 2.1. Materials

**Figure 1:**
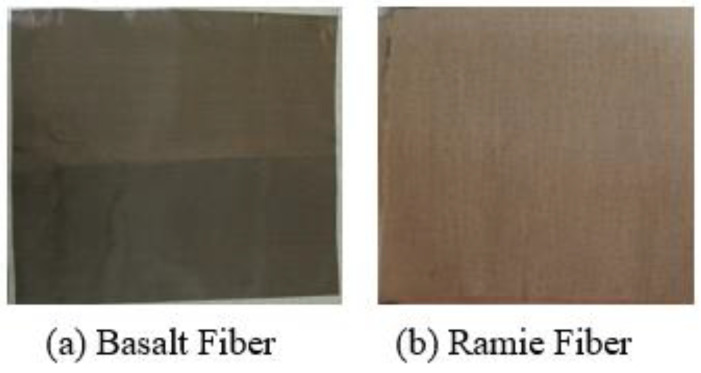
(**a**) Basalt (**b**) Ramie reinforcement for composite fabrication.

For this investigation, 400 GSM woven basalt and a ramie fabric mat with fiber random orientation direction were used to enhance the mechanical properties through the addition of fibers. These materials were purchased from GO Green Products Pvt. Ltd., Chennai, India. Although polyester matrix has a low density and low cost, similar to an epoxy matrix, polyester and an acceleration catalyzer were used for curing purposes. The physical characterization of basalt and ramie fibers’ physical properties depends on the manufacturing process and additions [25]. Table 1 provides the various properties of basalt and ramie fibers, as well as matrix polyester [26,27]. Figure 1 shows the basalt and ramie fibers used as reinforcement for composite fabrication.

The catalyzer used in this study is an aliphatic primary amine, while the polyester resin is bi-functional. Saturated Polyester Resin (Brand—Adarsh Fibre Pvt. Ltd., New Delhi, India) in liquid form was used. In most cases, the polyester is premixed and homogenized with the accelerator and catalyzer. Natural fibers and polyester matrices have good bonding between fibers and the matrix. General Purpose Polyester (Brand—Adarsh Fibre Pvt. Ltd., New Delhi, India) was used as a catalyst and accelerator, with a matrix ratio of 10:1 resin and a hardener that served as a binder and cured the hybrid composite. 

### 2.2. Preparation of Composite Laminates

**Figure 2:**
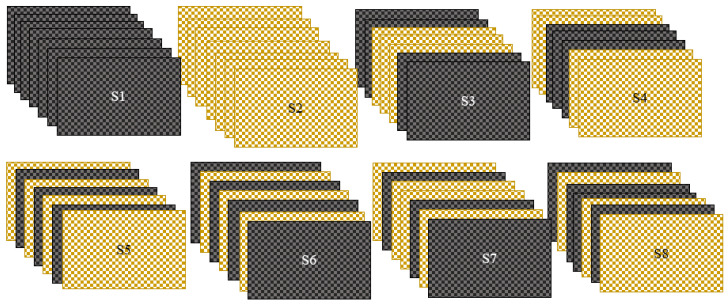
Stacking sequence of composite laminates. (S1-BBBBBBBB; S2-RRRRRRR; S3-BBRRRBB; S4-RRBBBRR; S5-BRRBRRB; S6-RBBRBBR; S7-BRBRBRB; S8-RBRBRBR).

Various laminates were fabricated, as shown in Table 2, and the polyester resin and accelerator catalyzer mix were prepared in a separate setup. The specimens were then covered with a plastic cover and sealed with sealant tape to prevent leakages. One end of the corner was attached to a vacuum pump, which was used to remove excess resin from the mold. A pressure of 2.04 kg/cm^2^ was maintained for 8–10 min, after which the composite laminate was opened and hot air was used to dry it under wet-to-dry conditions. The laminate was then post-cured for 24 h while maintaining a weight of 25 kg. Figure 2 shows the stacking sequence of composite laminates [28,29]. 

**Figure 3:**
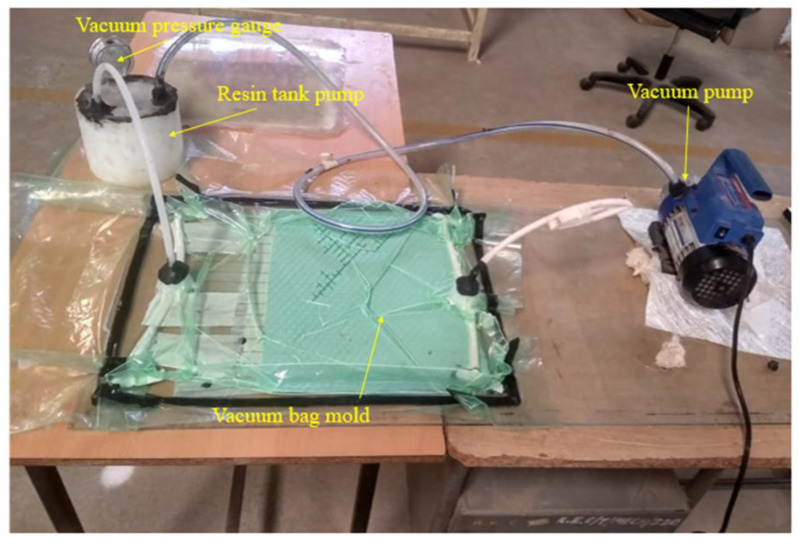
Composite materials are fabricated by vacuum bagging techniques.

As seen in Figure 3, the vacuum bag infusion technique is commonly used when working with composite materials. Hybrid composite laminates with different layers of unidirectional basalt and ramie reinforcement were created using a vacuum bag infusion approach that followed fiber stacking sequence techniques. First, clean polyester basalt and pure polyester ramie laminates were made as neat laminates, and the remaining six hybrid composites were prepared by varying the cross-ply of both fibers [26]. The bottom surface of the vacuum bag infusion setup was glass with a releasing agent added.

Design calculation of hybrid composites: The size of the fibers is 25 × 25 cm^2^.

Hybrid sample specimen 1:

This sequence consists of BRBRBRB (4B + 3R) mats.

4 Basalt fiber weight = 91.02 g.

3 Ramie fiber weight = 54.90 g.

Total reinforcement weight = 91.02 + 54.90 = 145.92 g.

Resin weight = 145.92 × 1.5 = 218.88 g (1:1.5 ratio of fiber weight).

Hardliner weight = 21.88 g (10:1 ratio of resin weight).

### 2.3. Mechanical Characterization Test

Hardness, tensile strength, flexural strength and impact energy are some of the mechanical parameters that were evaluated for the manufactured composite laminates, including the six hybrids. Coupon specimens were prepared following ASTM requirements for testing. The tests for hardness, tensile strength, flexural strength and impact were carried out with the help of ASTM D2240, D638, D790 and D256, respectively. Tensile (165 × 19 × 3 mm), flexural (100 × 12.7 × 3 mm), compression test (12.7 × 12.7 × 25.4 mm), hardness (30 × 30 mm) and impact (66.5 × 12.7 × 3 mm) samples were prepared according to ASTM standards. The specimens were cut to the specified dimensions for analysis.

**Figure 4:**
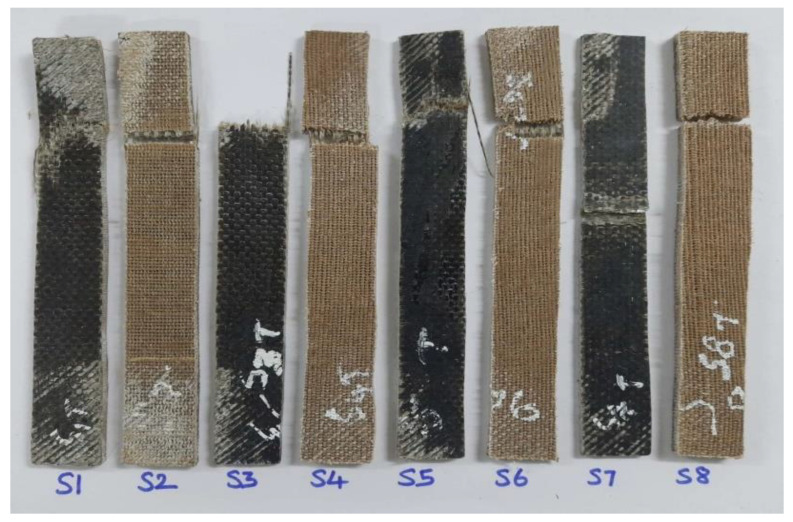
Tensile specimen after the test. (S1-BBBBBBBB; S2-RRRRRRR; S3-BBRRRBB; S4-RRBBBRR; S5-BRRBRRB; S6-RBBRBBR; S7-BRBRBRB; S8-RBRBRBR).

**Figure 5:**
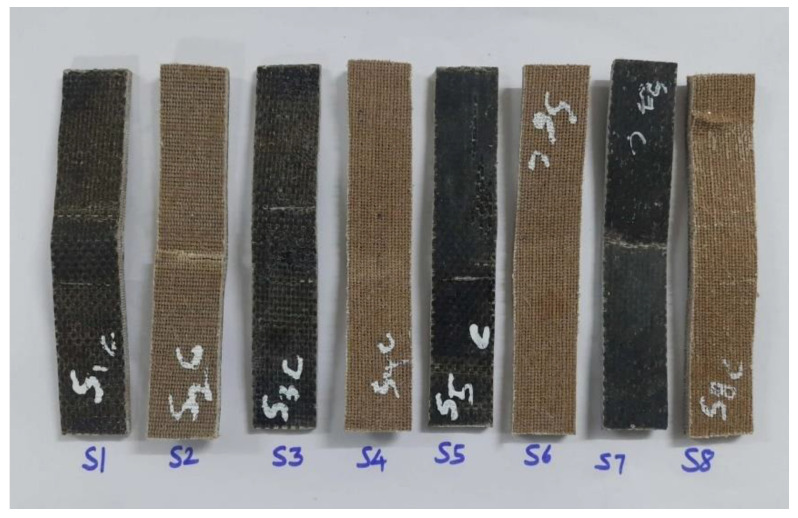
Flexural specimen after the test. (S1-BBBBBBBB; S2-RRRRRRR; S3-BBRRRBB; S4-RRBBBRR; S5-BRRBRRB; S6-RBBRBBR; S7-BRBRBRB; S8-RBRBRBR).

**Figure 6:**
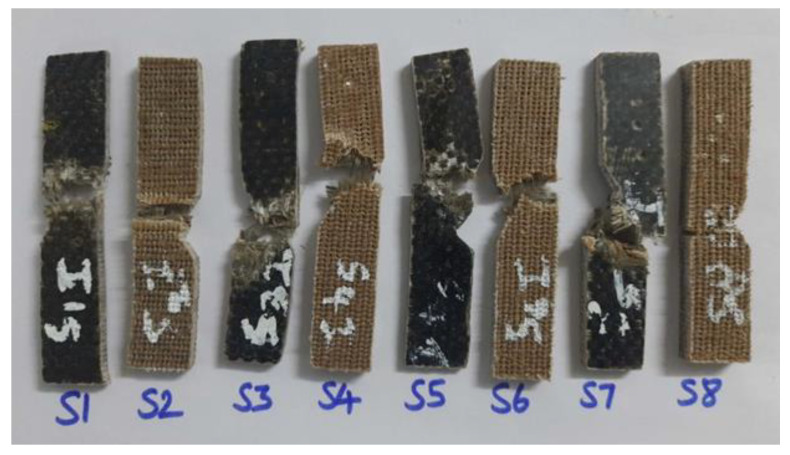
Impact specimen after the test. (S1-BBBBBBBB; S2-RRRRRRR; S3-BBRRRBB; S4-RRBBBRR; S5-BRRBRRB; S6-RBBRBBR; S7-BRBRBRB; S8-RBRBRBR).

SEM was used to examine the broken surfaces of the tested specimens. The scratched-up skin of the mechanical specimens was subjected to scanning electron microscope examination. The test’s purpose was to determine not only the quality of the material but also how it failed when subjected to the applied load. Figure 4 depicts the tensile test specimens used in this study to establish the mechanical characteristics of the material. The mixed laminate combination was designated as S2, S3, S4, S5, and S6, while the two remaining composite laminate configurations were designated as S1 and S2, respectively. The Izod impact test equipment was used to compare the mechanical characteristics of the various configurations. The surface of mechanically tested specimens that had been broken was the target of scanning electron microscope inspection. The test’s purpose was to determine not only the quality of the material but also how it failed when subjected to the applied load. Figure 5 and Figure 6 depict the flexural test specimens and impact test specimens used in this study.

**Figure 7:**
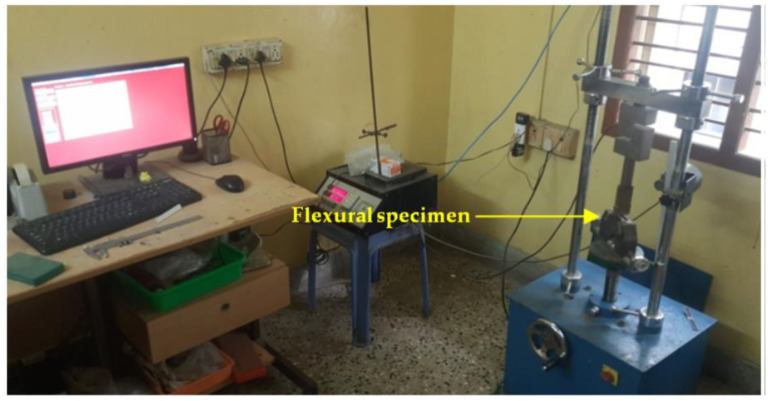
A universal testing machine was used to conduct the experiments.

On the same equipment, tensile tests, compression tests, and flexural testing were carried out by changing the grips to a three-point bending arrangement, as shown in Figure 7. A UTM machine (FIE-Blue Star, Kolhapur, MH, India; Cap.0–100 kN, Model: Instron-UNITEK-94100) was used. The Izod impact testing machine and the Shore D hardness testing equipment were used to determine the impact energy and hardness of the produced composite laminates. Three specimens were selected for each mechanical characterization trial, and the average of the three is provided as the mechanical property value in this research. When comparing the mechanical characteristics of the different laminate configurations, the hybrid laminate combinations were designated as S3, S4, S5, S6, S7, and S8. In contrast, the two remaining composite laminate configurations were designated as S1 and S2, respectively [28,29,30].

## 3. Results and Discussion

### 3.1. Tensile Properties of Hybrid Composites

**Figure 8:**
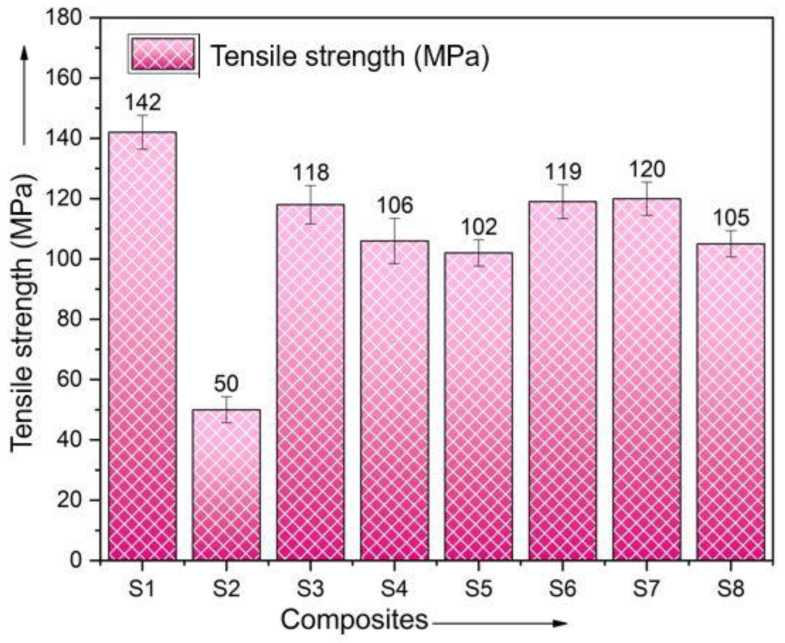
Comparison of tensile strength.

To assess the effect of vacuum bag molding on the strength of the composites, the tensile strength of mats made from composites including basalt and ramie fibers as reinforcing materials was examined. The mats were stacked in a variety of different sequences. Figure 8 illustrates how the tensile strength of produced composite laminates can vary depending on the sequencing patterns used. Compare the tensile strength of each sequencing combination with that of pure basalt and ramie fibers. Among the other possible combinations, the composite made with the BRBRBRB sequence arrangement was found to have the highest tensile strength of 120 MPa.

The tensile strength exhibited by the hybrid composites is related to the load transmission along the fibers. In this study, woven fiber mats were used as reinforcement materials. These allow the load to be distributed along the lateral and longitudinal directions of the loading. Therefore, the produced hybrid composites show an anisotropic nature, which significantly contributes to their tensile strength.

Figure 8 compares the tensile strengths of the six hybrid composite laminates, as well as the two plain composite laminates, that were used in this study. Out of the six hybrid composites of different composites tested specimens, the single fiber showed the lowest tensile strength, which ranged from a minimum of 102 MPa to a maximum of 120 MPa. Out of the eight specimens, the hybrid composite laminate made from the S7 material achieved a tensile strength of 120 MPa, the highest value achieved by any of the laminates. The hybrid laminate S2 had the minimum tensile strength compared to all the specimens at 50 MPa. The remaining hybrid laminates, such as S3, S4, S5, S6, and S8, each have tensile strengths of 118 MPa, 106 MPa, 102 MPa, 119 MPa, and 105 MPa, respectively. Pure basalt and polyester (S1) and ramie and polyester (S2) achieved tensile strengths of 142 and 50 MPa, respectively, when tested. Compared to pure laminates, hybrid composite laminates have superior mechanical properties [31,32].

**Figure 9:**
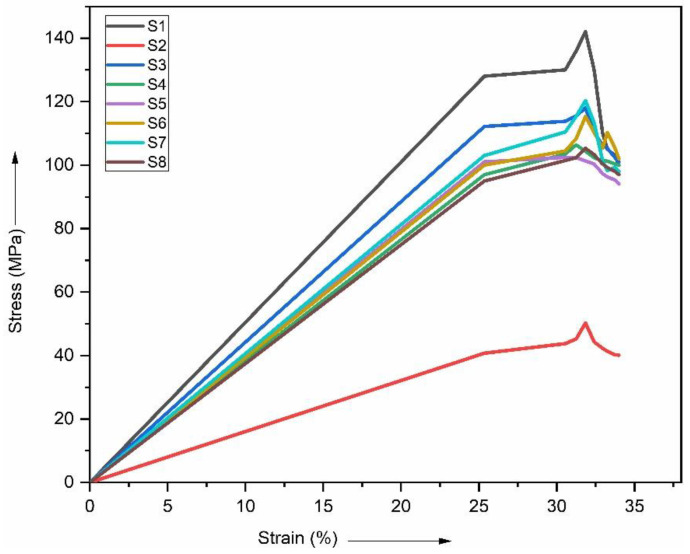
Stress vs. strain of tensile strength.

Figure 9 shows the stress–strain curve of the various composite laminates tested. From the graphs, it can be seen that as stress increases, strain decreases. The neat or plain samples show the minimum and maximum values, while the remaining six samples show average values. The hybrid sample S7 produces a tensile strength of 120 MPa. However, it is thought that pure laminates have mechanical properties that fall somewhere in between the two. The same can be seen in this study, with the difference that the hybrid laminate S7 achieved the highest tensile strength out of all of the materials tested. This finding should be treated as an outlier, and further testing is needed to confirm this behavior.

### 3.2. Flexural Properties of Produced Composites

**Figure 10:**
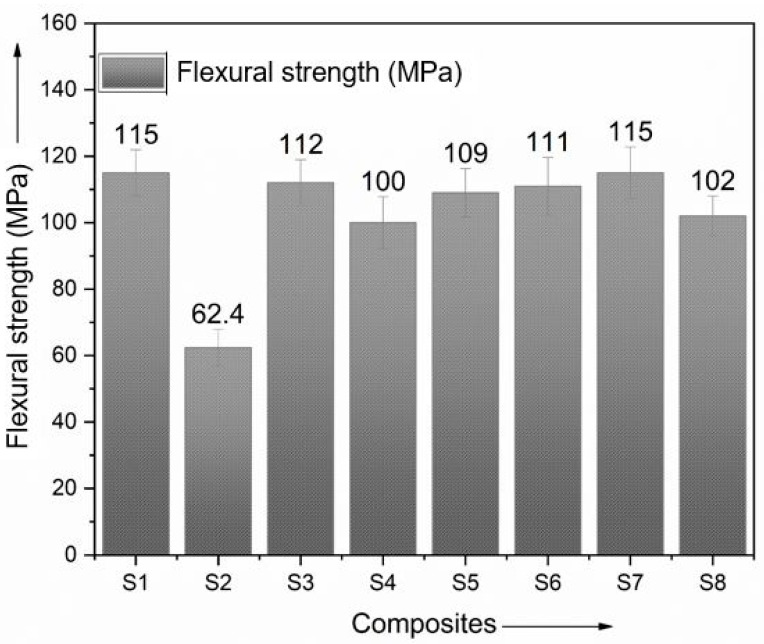
Comparison of flexural strength.

According to the investigation’s findings, the hybrid composites that contained ramie fiber sandwiched between polyester had a maximum flexural strength of 115 MPa, with a minimum of 100 MPa. The least amount of flexural strength was achieved using pure ramie fiber for the polyester. The stacking sequence (BRBRBRB) was found to meet the surface strength and endurance requirements. The outer layer of ramie contributed to an increase in both bending stress and elastic modulus. Figure 10 shows the test specimens’ values after flexural testing, which reveals that the sequence in which the reinforcing fibers were inserted into the composite materials significantly influenced the composite’s characteristics. When the stacking order of the reinforcing material was changed, there was a noticeable improvement in the flexural strength of the hybrid composites, as demonstrated by the results obtained during the loading of the flexural specimens.

In addition, the arrangement of the woven fibers in a bidirectional manner helped to distribute the applied load uniformly across the layers, which increased its capacity to bear the weight placed on it from the outside [33,34].

### 3.3. Compression Strength of Produced Composites

**Figure 11:**
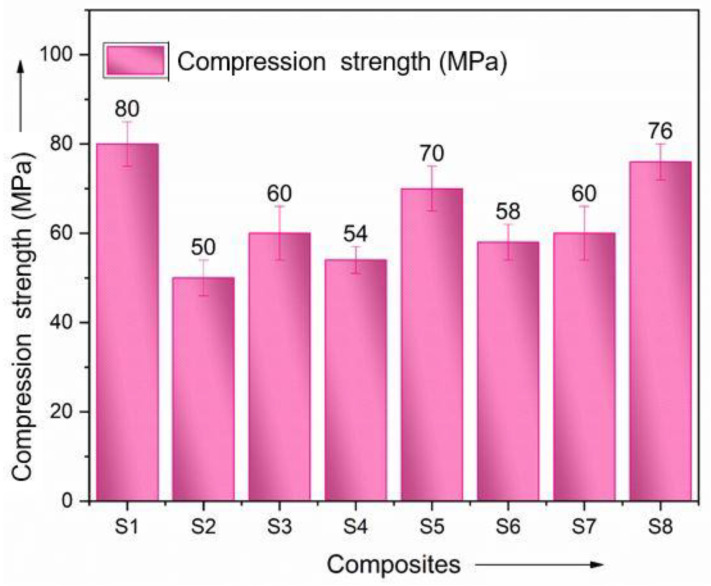
Comparison of compression strength.

The compression strength of all six hybrid composites was only able to be increased to a limited extent, ranging from 50 MPa to 76 MPa. The use of consecutive layers of the same reinforcing fibers helped improve compression strength by increasing stiffness, which was achieved through layering. Interestingly, the plain laminate S1 and the hybrid laminate S7, which contained all seven layers of basalt fibers and alternatingly stacked ramie fibers sandwiching the basalt fibers, both showed poorer compression characteristics. S2 was the simplest of the three laminates. The compressive state of the hybrid composite laminates is shown in Figure 11.

The matrix material, effectively bonding with the basalt fibers against the ramie fibers, is credited with this. The pure and hybrid laminate designs in this study behaved similarly in the flexural strength tests [35,36].

### 3.4. Impact Energy of Produced Composites

**Figure 12:**
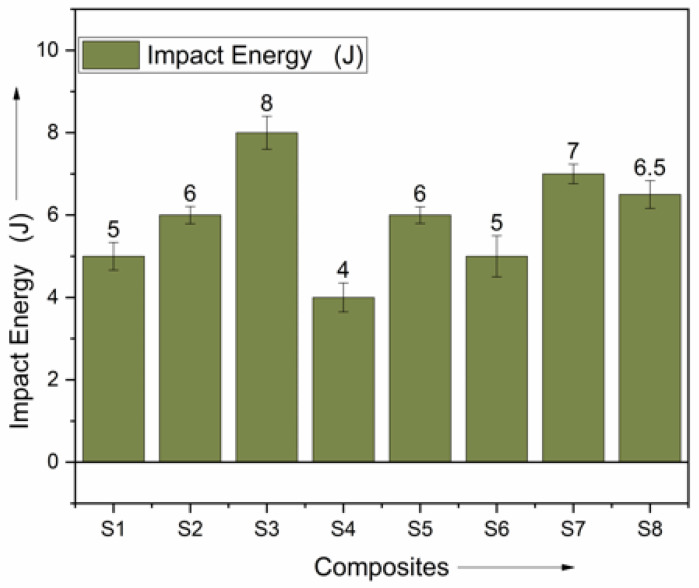
Comparison of impact energy.

An izod impact energy test was carried out on the fabricated hybrid composite materials. The varying levels of impact energy are shown in Figure 12. Composite materials ranging from S3 to S8 only produced modest impact energy compared to S1. It is believed that stacking by a variety of the same reinforcing fibers increases stiffness, which helps improve impact energy. This can be achieved by layering the fibers in successive layers. Impact resistance is increased in the plain laminate S2 and composite laminate S1, which consist of five layers of ramie strands and alternatingly stacked basalt fibers. The hybrid composite known as S8, consisting of alternating layers of natural fiber sandwiching ramie fibers, was able to absorb the greatest amount of energy, equal to 7 J, as determined by comparing the relative energies of the various composites. Its counterparts, classified as S1 and consisting of alternating layers of basalt strands sandwiched between two basalt fibers, had a stronger impact energy and were able to absorb 5 J of impact energy.

For comparison, plain basalt and ramie fiber laminates provided the minimum impact energy, while hybrid composite laminates produced the highest impact energy by altering the composite of (RBRBRBK) and a polyester matrix.

### 3.5. Hardness of Produced Composites

**Figure 13:**
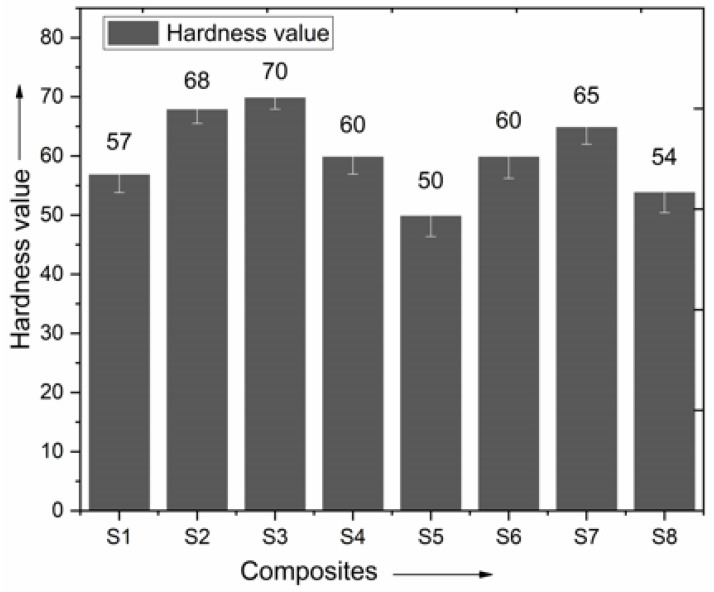
Comparison of hardness.

Figure 13 shows the hardness of the two basic composite laminates and six hybrid laminates made solely from basalt and ramie fibers for this study. Of the 34 samples tested, the one with the softest Shore-D hardness was reinforced with seven layers of ramie fibers (S2). In contrast, its S2 counterpart, which has all seven layers and basalt fibers, is 68-hard. As a result, the maximum number of stacking sequences for hybrid composites was 77.

Ramie fiber has a higher hardness absorption rate than basalt fiber. However, hybrid composites made from S3, S4, S5, S6, S7, and even S8 may be hardened to levels equivalent to S7. The results show that hybridization influences the hardness of the resulting composite laminates.

### 3.6. Microstructural Analysis of Hybrid Composites

After the tensile test, SEM micrographs of hybrid composites were obtained. SEM analysis was carried out using TESCAN VEGA 3 microscope at the Centre for Nanoscience and Technology, Anna University, Chennai, India.

**Figure 14:**
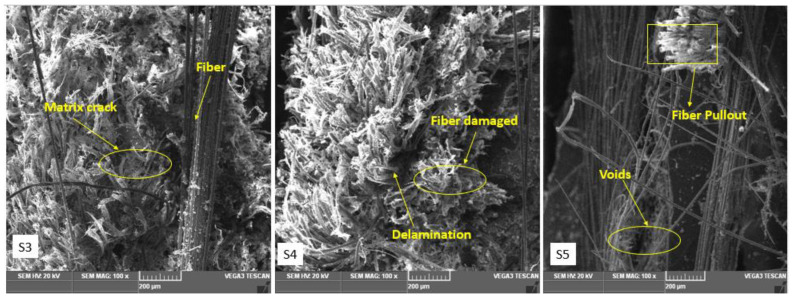

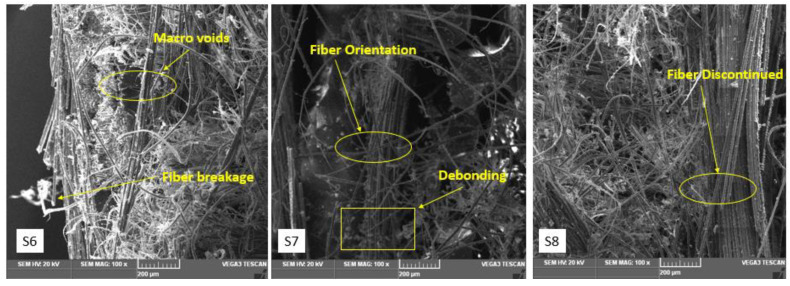
SEM images of S3, S4, S5, S6, S7 and S8 tensile fracture specimens.

According to the SEM examination, the matrix element had strong bonding with the reinforcing fibers. However, under the impact of the tensile load, the fibers were drawn out. As a result, the reinforcing fibers were pushed out by the matrix and the tensile load fractured in a brittle manner. After conducting tensile experiments, failure samples were subjected to a microstructural study. The surface morphology of a composite specimen subjected to a tensile fracture is shown in Figure 14, as seen by scanning electron microscopy. The hybrid composites were analyzed with a universal testing machine (UTM), which applied a constant force to test specimens to detect failure mechanisms such as matrix cracking, fiber breakage, fiber pull-out from a matrix, and debonding between the two [37,38,39]. Fiber breakage occurred as a result of an increase in transverse loading. There is no filler material in the matrix, yet the load transfer is the same for specimens 1 and 5. In this research, 60% of fiber composites performed poorly in terms of balancing and distributing the load. Matrix cracks caused by transverse loads are similar to those caused by uneven additive filler distribution in a laminate sample. The photos show visible micro-voids, damaged fibers, and fibers in another area, as well as the composites.

These factors diminished tensile strength and stiffness. However, the polyester matrix allowed the hybrid fibers to improve mechanical characteristics despite the loss of hygroscopicity in the tissues. The SEM analysis also found that the micropores were a significant cause of the damage when stress was applied.

### 3.7. Fractographical Image of Flexural Fracture Specimens

**Figure 15:**
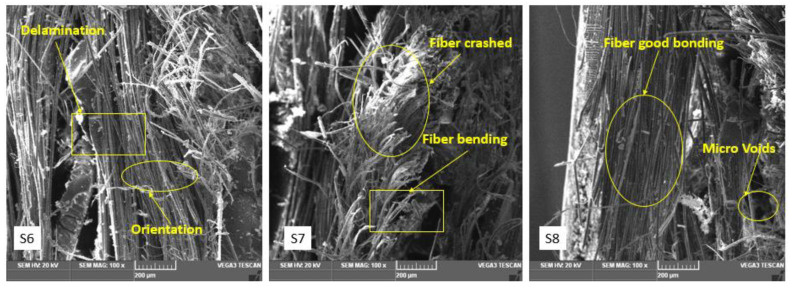
SEM images of S6, S7 and S8 flexural fracture composites specimens.

Three-point bending tests were performed to study flexural behavior. Failure of the specimens was observed as debonding and delamination of an interfacial region of the fibers and matrix, since the load was applied progressively. Figure 15 shows the SEM micrographs for flexural fracture composite specimens. The images show that the fibers under flexure created cavities in another region. This reduced the hybrid composite’s ability to withstand flexural loads.

The SEM images in Figure 15 show a bending test with transverse and compression force on the upper portion of the hybrid laminated composites. It was observed that the flexural properties of the composite provide the same trend as the tensile characterization of composites. The sample specimens S6 and S7 showed low failure damages. The scientific reason behind this is the minimal formation of SiC additive particulates. The microstructure image of fracture surfaces reveals shear stress in the center layer surface of the laminate specimens. The failure mechanism of voids, matrix cracks, fiber pullout and fiber breakage is due to the release of the additive particle when an applied load is present [40,41].

## 4. Conclusions

The present study reported on the influence of hybridization on the tensile, flexural, impact, hardness, and compression response of hybrid composites. Eight hybrid composite laminates were fabricated with polyester as a matrix, and basalt/ramie fiber was reinforced using the vacuum bag infusion method. According to ASTM standards, tensile, flexural, impact, compression, and hardness tests were performed on these produced laminates. In the tensile and compression tests, the hybrid composite of sample 1 (BRBRBRB) had a higher tensile strength of 120 MPa and flexural strength of 115 MPa, as well as a higher compression strength of 76 MPa and improved Young’s modulus compared to other composite materials. In the flexural test, the hybrid composite of sample 7 (BBRRRBB) had a higher flexural strength of 115 MPa than other composite materials. This stacking sequence could be used for functional parts such as car doors. In the impact test, the hybrid composite of sample three (BBRBRBB) had a higher impact energy of 8 J compared to other compositions. In the Shore D hardness test, the composite of sample two (RBRBRBR) had a high hardness of 76 MPa compared to other composites. This hybrid composite S6 is suitable for producing automobiles and panels, and building walls that demand a balance of mechanical properties.

## Figures and Tables

**Table 1:** Physical characteristics of materials.

Fibers	Strength (MPa)	Modulus Elasticity (GPa)	$\mathrm{Density}\;(g/m^{3})$	Poisson’s Ratio	Type
Basalt (B)	310–420	110	2.8	0.24	Woven
Ramie (R)	180–205	80	1.2	0.2	Woven
Polyester	40	3	1.09	0.23	Resin

**Table 2:** Compositions of specimens neatly arranged.

Code	Stacking Structure Fiber	Volume Fraction of Matrix %	Fiber Volume Fraction %
S1	BBBBBBBB	45% polyester	55%
S2	RRRRRRR		
S3	BBRRRBB		
S4	RRBBBRR		
S5	BRRBRRB		
S6	RBBRBBR		
S7	BRBRBRB		
S8	RBRBRBR		

B-Basalt Fiber; R-Remie Fiber.

## Data Availability

The data presented in this study are available through email upon request to the corresponding author.

## References

[1] Huang Y., Zhang W., Liu X. (2022). Assessment of Diagonal Macrocrack-Induced Debonding Mechanisms in FRP-Strengthened RC Beams. J. Compos. Constr..

[2] Shcherbakov A., Mostovoy A., Bekeshev A., Burmistrov I., Arzamastsev S., Lopukhova M. (2022). Effect of microwave irradiation at different stages of manufacturing unsaturated polyester nanocomposite. Polymers.

[3] Shi M., Wang R., Li L., Chen N., Xiao P., Yan C., Yan X. (2022). Redox-active polymer integrated with mxene for ultra-stable and fast aqueous proton storage. Adv. Funct. Mater..

[4] Rahman M., Hoque A., Gafur M., Khan R.A., Hossain M.K. (2019). Study on the mechanical, electrical and optical properties of metal-oxide nanoparticles dispersed unsaturated polyester resin nanocomposites. Results Phys..

[5] Ganesan V., Shanmugam V., Kaliyamoorthy B., Sanjeevi S., Shanmugam S., Alagumalai V., Krishnamoorthy Y., Försth M., Sas G., Razavi S.J. (2021). Optimisation of mechanical properties in saw-dust/woven-jute fibre/polyester structural composites under liquid nitrogen environment using response surface methodology. Polymers.

[6] Deng E.-F., Zhang Z., Zhang C.-X., Tang Y., Wang W., Du Z.-J., Gao J.-P. (2023). Experimental study on flexural behavior of UHPC wet joint in prefabricated multi-girder bridge. Eng. Struct..

[7] Karthik K., Rajamani D., Manimaran A., Udayaprakash J. (2020). Evaluation of tensile properties on Glass/Carbon/Kevlar fiber reinforced hybrid composites. Mater. Today Proc..

[8] Mohanavel V., Kumar S.S., Vairamuthu J., Ganeshan P., NagarajaGanesh B. (2021). Influence of stacking sequence and fiber content on the mechanical properties of natural and synthetic fibers reinforced penta-layered hybrid composites. J. Nat. Fibers.

[9] Yoganandam K., Ganeshan P., NagarajaGanesh B., Raja K. (2019). Characterization studies on Calotropis procera fibers and their performance as reinforcements in epoxy matrix. J. Nat. Fibers.

[10] Kalita K., Ghadai R.K., Bansod A. (2022). Sensitivity analysis of gfrp composite drilling parameters and genetic algorithm-based optimisation. Int. J. Appl. Metaheuristic Comput..

[11] Nagamadhu M., Jeyaraj P., Kumar G.C.M. (2020). Influence of textile properties on dynamic mechanical behavior of epoxy composite reinforced with woven sisal fabrics. Sādhanā.

[12] Abhilash S., Singaravelu D.L. (2020). A comparative study of mechanical, dynamic mechanical and morphological characterization of tampico and coir fibre-reinforced LLDPE processed by rotational moulding. J. Ind. Text..

[13] Ramesh V., Anand P. (2020). Evaluation of mechanical properties on Kevlar/Basalt fiber reinforced hybrid composites. Mater. Today Proc..

[14] Sun G., Tong S., Chen D., Gong Z., Li Q. (2018). Mechanical properties of hybrid composites reinforced by carbon and basalt fibers. Int. J. Mech. Sci..

[15] Sapuan S.M., Aulia H.S., Ilyas R.A., Atiqah A., Dele-Afolabi T.T., Nurazzi M.N., Supian A.B.M., Atikah M.S.N. (2020). Mechanical properties of longitudinal basalt/woven-glass-fiber-reinforced unsaturated polyester-resin hybrid composites. Polymers.

[16] Han Q., Shi S., Liu Z., Han Z., Niu S., Zhang J., Qin H., Sun Y., Wang J. (2020). Study on impact resistance behaviors of a novel composite laminate with basalt fiber for helical-sinusoidal bionic structure of dactyl club of mantis shrimp. Compos. Part B Eng..

[17] Chaudhary V., Bajpai P.K., Maheshwari S. (2018). An investigation on wear and dynamic mechanical behavior of jute/hemp/flax reinforced composites and its hybrids for tribological applications. Fibers Polym..

[18] Behera R.R., Ghadai R.K., Kalita K., Banerjee S. (2016). Simultaneous prediction of delamination and surface roughness in drilling GFRP composite using ANN. Int. J. Plast. Technol..

[19] Karthik K., Senthilkumar P. (2015). Tribological Characteristics of Carbon-Epoxy with Ceramic Particles Composites for Centrifugal Pump Bearing Application. Int. J. ChemTech Res..

[20] Kalita K., Mallick P.K., Bhoi A., Ghadai K. (2018). Optimizing drilling induced delamination in gfrp composites using genetic algorithm& particle swarm optimisation. Adv. Compos. Lett..

[21] Quagliarini E., Monni F., Bondioli F., Lenci S. (2016). Basalt fiber ropes and rods: Durability tests for their use in building engineering. J. Build. Eng..

[22] Prasad V.V., Talupula S. (2018). A review on reinforcement of basalt and aramid (kevlar 129) fibers. Mater. Today Proc..

[23] Das S.C., Paul D., Grammatikos S.A., Siddiquee A., Papatzani S., Koralli P., Islam J.M., Khan M.A., Shauddin S., Khan R.A. (2021). Effect of stacking sequence on the performance of hybrid natural/synthetic fiber reinforced polymer composite laminates. Compos. Struct..

[24] Khalid M., Peng Q. (2021). Sustainability and environmental impact of additive manufacturing: A literature review. Comput. Des. Appl..

[25] TG Y.G., Kushvaha V., MR S., Siengchin S. (2021). A new study on flax-basalt-carbon fiber reinforced epoxy/ bioepoxy hybrid composites. Polym. Compos..

[26] Srivathsan A., Vijayaram B., Ramesh R. (2017). Gokuldass investigation on mechanical behavior of woven fabric glass/kevlar hybrid composite laminates made of varying fibre inplane orientation and stacking sequence. Mater. Today Proc..

[27] Raja T., Mohanavel V., Kumar S.S., Rajkumar S., Ravichandran M., Subbiah R. (2021). Evaluation of mechanical properties on kenaf fiber reinforced granite nano filler particulates hybrid polymer composite. Mater. Today Proc..

[28] Fragassa C., Pavlovic A., Santulli C. (2018). Mechanical and impact characterisation of flax and basalt fibre vinylester composites and their hybrids. Compos. Part B Eng..

[29] Dhuban S.B., Karuppanan S., Mengal A.N., Patil S.S. (2017). Effect of fiber orientation and ply stacking sequence on buckling behaviour of basalt-carbon hybrid composite laminates. Indian J. Eng. Mater. Sci..

[30] Thandavamoorthy R., Palanivel A. (2019). Testing and evaluation of tensile and impact strength of neem/banyan fiber-reinforced hybrid composite. J. Test. Eval..

[31] Thooyavan Y., Kumaraswamidhas L., Raj R.E., Binoj J. (2021). Influence of SiC micro and nano particles on tribological, water absorption and mechanical properties of basalt bidirectional mat/vinyl ester composites. Compos. Sci. Technol..

[32] Raja T., Anand P., Sundarraj M., Karthick M., Kannappan A. (2018). Failure analysis of natural fiber reinforced polymer composite leaf spring. Int. J. Mech. Eng. Technol..

[33] Karthik K., Prakash J.U., Binoj J.S., Mansingh B.B. (2022). Effect of stacking sequence and silicon carbide nanoparticles on properties of carbon/glass/Kevlar fiber reinforced hybrid polymer composites. Polym. Compos..

[34] Ramesh V., Karthik K., Arunkumar K., Unnam N.K., Ganesh R., Rajkumar C. (2022). Effect of sawdust filler with Kevlar/basalt fiber on the mechanical properties epoxy–based polymer composite materials. Mater. Today Proc..

[35] Shcherbakov A.S., Mostovoy A.S., Yakovlev N.A., Arzamastsev S.V. (2021). Effect of carbon nanotube functionalization on the physicochemical and mechanical properties of modified fiber-reinforced composites based on an epoxy resin. Russ. J. Appl. Chem..

[36] Shahid M., Farooqi Z.H., Begum R., Arif M., Irfan A., Azam M. (2020). Extraction of cobalt ions from aqueous solution by microgels for in-situ fabrication of cobalt nanoparticles to degrade toxic dyes: A two fold-environmental application. Chem. Phys. Lett..

[37] Arif M., Farooqi Z.H., Irfan A., Begum R. (2021). Gold nanoparticles and polymer microgels: Last five years of their happy and successful marriage. J. Mol. Liq..

[38] Begum R., Naseem K., Farooqi Z.H. (2015). A review of responsive hybrid microgels fabricated with silver nanoparticles: Synthesis, classification, characterization and applications. J. Sol-Gel Sci. Technol..

[39] Shahid M., Farooqi Z.H., Begum R., Arif M., Azam M., Irfan A., Farooq U. (2021). Multi-functional organic–inorganic hydrogel microspheres as efficient catalytic system for reduction of toxic dyes in aqueous medium. Zeitschrift für Physikalische Chemie.

[40] Arif M. (2022). Complete life of cobalt nanoparticles loaded into cross-linked organic polymers: A review. RSC Adv..

[41] Shahid M., Farooqi Z.H., Begum R., Arif M., Wu W., Irfan A. (2019). Hybrid microgels for catalytic and photocatalytic removal of nitroarenes and organic dyes from aqueous medium: A review. Crit. Rev. Anal. Chem..

